# Genetic factors influencing risk to orofacial clefts: today’s challenges and tomorrow’s opportunities

**DOI:** 10.12688/f1000research.9503.1

**Published:** 2016-11-30

**Authors:** Terri H. Beaty, Mary L. Marazita, Elizabeth J. Leslie

**Affiliations:** 1 Department of Epidemiology, Johns Hopkins University, Baltimore, MD, 21205, USA; 2 Department of Oral Biology and Center for Craniofacial and Dental Genetics, University of Pittsburgh, Pittsburgh, PA, 15219, USA

**Keywords:** orofacial clefts, CHD1, ADAMTS9, whole genome sequencing

## Abstract

Orofacial clefts include cleft lip (CL), cleft palate (CP), and cleft lip and palate (CLP), which combined represent the largest group of craniofacial malformations in humans with an overall prevalence of one per 1,000 live births. Each of these birth defects shows strong familial aggregation, suggesting a major genetic component to their etiology. Genetic studies of orofacial clefts extend back centuries, but it has proven difficult to define any single etiologic mechanism because many genes appear to influence risk. Both linkage and association studies have identified several genes influencing risk, but these differ across families and across populations. Genome-wide association studies have identified almost two dozen different genes achieving genome-wide significance, and there are broad classes of ‘causal genes’ for orofacial clefts: a few genes strongly associated with risk and possibly directly responsible for Mendelian syndromes which include orofacial clefts as a key phenotypic feature of the syndrome, and multiple genes with modest individual effects on risk but capable of disrupting normal craniofacial development under the right circumstances (which may include exposure to environmental risk factors). Genomic sequencing studies are now underway which will no doubt reveal additional genes/regions where variants (sequence and structural) can play a role in controlling risk to orofacial clefts. The real challenge to medicine and public health is twofold: to identify specific genes and other etiologic factors in families with affected members and then to devise effective interventions for these different biological mechanisms controlling risk to complex and heterogeneous birth defects such as orofacial clefts.

## Introduction

**Figure 1.**
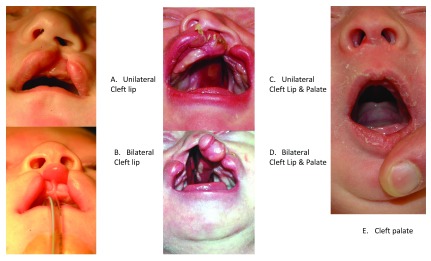
Examples of orofacial clefts. **A**. Left unilateral cleft lip. **B**. Bilateral cleft lip. **C**. Left unilateral cleft lip plus cleft palate. **D**. Bilateral cleft lip plus cleft palate. **E**. Cleft palate.

Orofacial clefts represent a group of anatomically distinct birth defects where there is a gap or break in normal features of the mouth, most commonly the roof of the mouth (the palate) or the upper lip or both. As shown in Figure 1, the most common anatomical forms of orofacial clefts include cleft lip (CL), cleft palate (CP), and cleft lip and palate (CLP). Other features of the face and mouth can also be affected by orofacial clefts, but the overwhelming majority of all cases are infants with one of these three common forms ^1^. Normally, development of the face and mouth occurs very early in pregnancy and reflects a complex process of cell growth and migration followed by fusion of symmetric structures to form the palate separating the mouth and nasal cavity, with the outer structures of the face developing before the inner structures. Collectively CL, CLP, and CP are the most common craniofacial birth defects worldwide, and affected individuals face feeding difficulties early in life and typically require multiple corrective surgeries, therapeutic dental procedures, and speech therapy throughout childhood ^2– 4^. In addition, individuals born with an orofacial cleft have increased incidence of mental health problems and higher overall mortality rates at all stages of life, even in developed countries with good medical care ^5, 6^. Where access to medical care is severely limited, infants born with CP or CLP generally have high mortality rates due to difficulty in breastfeeding, while untreated CL and CLP cases can face social discrimination throughout their lives. Thus, these birth defects have been subject to substantial selective pressure for most of human history.

Although clefts develop early in pregnancy, most epidemiologic data are based on cleft frequencies at birth. The worldwide average birth prevalence of all orofacial clefts is 9.92 per 10,000 (close to one per 1,000), but there is substantial variation among populations ^7^. In general, East Asian and Native American populations have substantially higher birth prevalence rates than do European and South Asian populations, while African ancestry populations have lower birth prevalence rates ^8– 10^.

### Environmental risk factors for orofacial clefts

There are several recognized environmental risk factors and multiple genes involved in the etiology of orofacial clefts, which means the etiology is complex and heterogeneous. Maternal smoking during pregnancy is a recognized risk factor for orofacial clefts, and it is estimated that 6.1% (95% confidence interval [CI]=4.4–7.7%) of orofacial clefts could be avoided by eliminating maternal smoking ^11, 12^. Passive or “second-hand” exposure also appears to be a risk factor ^13^. Evidence for maternal alcohol consumption as a risk factor is less consistent, although consuming large amounts over a short period of time (e.g. “binge drinking”) appears to increase risk ^14^. Broad measures, e.g. socio-economic status, have been suggested as risk factors, but it is difficult to separate out the combined effects of maternal nutrition and health ^15^. Several prescription medications have also been reported to increase risk when taken during the first trimester, including folate antagonists and certain other drugs exhibiting anti-folate properties ^7^.

Interestingly, a substantial number of studies suggest genetic and environmental risk factors may “interact” to modify risk to orofacial clefts. Several common exposures, including maternal smoking, alcohol consumption, and vitamin supplementation (a protective factor), have yielded statistical evidence of gene–environment (GxE) interaction, although the results from different studies are not always consistent. While biologically GxE interaction is eminently rational, replicating statistical evidence across studies is difficult and proving interaction exists remains a major challenge ^16^.

### Familial aggregation and the genetic basis of orofacial clefts

Orofacial clefts show strong familial aggregation, which suggests a major genetic component to their etiology. Marazita and Leslie ^17^ have recently reviewed the extensive history of genetic studies of orofacial clefts, which dates back to the 18 ^th^ century (see their Table 1). Despite decades of genetic research, it remains unclear exactly how many genes might control risk or how they act to influence risk to orofacial clefts. In general, close relatives of cleft cases have a greatly increased risk of being affected. Nationwide vital records from Norway ^18^ show the relative risk to a first-degree relative of a case with CL with or without CP (CL/P, i.e. CL and CLP cases combined) was 32 times higher than the baseline population risk, and for relatives of a CP case this relative risk was 56 times higher than baseline risk. This increased risk drops with increasing genetic distance: nationwide registry data from Denmark spanning the entire second half of the 20 ^th^ century ^19^ show the relative risk among all first-degree relatives (parents, full siblings, and offspring) of CL/P cases was highest, while risks to second-degree relatives (half-siblings, avuncular relatives, and grandparents) were much smaller, and risks to third-degree relatives (first cousins and half-avuncular relatives) were not significantly above the baseline population risk in Denmark (see their Table 2). When rules of exclusions and phenotype definition were adjusted between these population-based studies (Norway and Denmark), both gave similar patterns of strong familial aggregation, i.e. relatives of cleft cases always had a high relative risk compared to the population’s baseline risk, and there is a steep drop with increasing genetic distance between relatives.

**Table 1.** Genes achieving genome-wide significance as influencing risk to orofacial clefts. Adapted and updated from Table 2 in Leslie and Marazita ^1^.

Associated locus	Candidate gene in region	Associated phenotype	Analysis method	References for genome-wide significance
1p36.13	*PAX7*	CL/P	GWAS meta-analysis, GWAS replication	36, 50, 51
1p36	*GRHL3*	CP	GWAS, GWAS replication	52, 53
1p22	*ARHGAP29*	CL/P	GWAS	36, 54
1q32.2	*IRF6*	CL/P	GWAS, linkage	25, 27, 33, 36, 54
2p13	*TGFA*	CL/P	Linkage	25
2p21	*THADA*	CL/P	GWAS meta-analysis	50
2p24	*FAM49A*	CL/P	GWAS	54
3p11	*EPHA3*	CL/P	GWAS meta-analysis	50
3q12	*COL8A1/FILIPIL*	CL/P	GWAS replication	51
3q27–28	*TP63*	CL/P	Linkage	25
8q21.3	*DCAF4L2*	CL/P	GWAS meta-analysis, GWAS replication	50, 51, 54
*8q22.3*	*BAALC*	CP & multivitamins	GWAS x E	55
*8q24*	Gene Desert	CL/P	GWAS	27, 33, 36, 54, 56
9q22.33	*FOXE1*	CL/P and CP	Linkage	25, 28
9q31.1	*SMC2*	CP & maternal alcohol	GWAS x E	55
10q25.3	*VAX1*	CL/P	GWAS	27, 36, 54
12q14	*TBK1*	CP & maternal smoking	GWAS x E	55
13q31.2	*SPRY2*	CLP	GWAS meta-analysis	50
14q21–24	*PAX9, TGFB3, BMP4*	CL/P	Linkage	25
15q22	*TPM1*	CL/P	GWAS meta-analysis	50
15q24	*ARID3B*	CL/P	GWAS	54
16p13	*CREBBP*	CL/P	GWAS	57
16q24	*CRISPLD2*	CL/P	Linkage	25
17p13.1	*NTN1*	CL/P	GWAS meta-analysis, GWAS replication	49– 51, 54
17q22	*NOG*	CL/P	GWAS	27, 49, 54
17q23.2	*TANC2*	CL/P	GWAS	54
18q22	*ZNF236*	CP & maternal smoking	GWAS x E	55
19q13.11	*RHPN2*	CL/P	GWAS	54
20q12	*MAFB*	CL/P	GWAS	36, 54

Abbreviations: CL/P, cleft lip with or without cleft palate; CLP, cleft lip and palate; CP, cleft palate; GWAS, genome-wide association study.

Twin studies of orofacial clefts consistently show higher concordance rates in monozygotic (MZ or identical) twins compared to dizygotic (DZ or fraternal) twins, again suggesting a major role for genes in controlling risk of being affected. Clearly, because MZ twins have identical genes over their entire genome, anything less than 100% concordance indicates some non-genetic component to the etiology of orofacial clefts, but whenever MZ twins consistently show greater concordance than DZ twins, it suggests some proportion of the variation in risk is under genetic control. Grosen *et al*. ^20^ linked population-level vital records (birth certificate and twin registry data from Denmark) spanning most of the 20 ^th^ century, achieving virtually complete ascertainment of cases for the latter part of the century. They showed MZ twins had much higher concordance rates than did DZ twins (47% MZ twins with 95% CI=31–64% compared to 8% for DZ twins with 95% CI=4–14%). These concordance rates yielded estimates of heritability (the proportion of variation in risk attributable to unobserved independent genes) of 91% for CL/P and 90% for CP. However, because the concordance rate among MZ twins is more than four times that of DZ twins, these Danish findings also raise the possibility that specific combinations of different genes (termed “epistasis”) may be important in the etiology of orofacial clefts.

While clearly orofacial cleft risk shows strong familial aggregation, it does not strictly adhere to Mendel’s laws of inheritance, even within multiplex families (i.e. those with two or more affected members), which has bedeviled geneticists for decades. Statistical methods for fitting genetic models to family data are collectively termed “segregation analyses”, and these trace back to the foundations of human genetics. Statistical methods were initially developed in the early 20 ^th^ century by statisticians such as R.A. Fisher, W. Weinberg, and J.B.S. Haldane ^17, 21^. By the 1970s, segregation analysis had evolved to allow formal tests within a framework of “mixed models” that simultaneously considered straightforward Mendelian components (i.e. a “major gene”) and a more general residual “heritability” reflecting combined effects of many independent genes (collectively termed “polygenes”). A number of segregation analyses of orofacial clefts were performed during the early 1970s through the early 1990s for various racial/ethnic groups ^21^. Collectively, however, this modeling approach rarely resulted in definitive and reproducible findings, and the most suitable model of inheritance remained the “multifactorial threshold model”, which is exquisitely vague in defining genetic mechanisms and statistically cannot discriminate between a single major gene vs. multiple “polygenes”. This multifactorial model hypothesizes a continuous “genetic liability” for risk, plus unspecified non-genetic risk factors that could also influence risk. Under this very general model, it is possible to estimate the “heritability” or the proportion of variation in risk attributable to any number of independent, autosomal genes, but it is not possible to estimate the penetrance or allele frequencies at any one of these separate risk genes.

Still, this multifactorial threshold model adequately explains the clear gender differences in risk to CL/P and CP (where the former shows a distinct excess of affected males, while the latter has a slight excess of females, suggesting gender-specific thresholds) and the higher risk to relatives of more severe cases (i.e. bilateral versus unilateral CL/P) ^19^. The sharp decline in relative risk to relatives of cases with increasingly distant relationships is also completely compatible with the multifactorial threshold model because the probability of sharing alleles identical by descent is constant whether one gene, a few genes, or many genes control risk. However, other explanations could potentially result in these same patterns of risk to relatives. Thus, even sophisticated statistical approaches to modeling genetic inheritance have failed to define any single biological mechanism controlling risk to orofacial clefts in all families; indeed it has been suggested that multiple genes, perhaps two to six independent genes (possibly up to 14) could determine risk to orofacial clefts ^22^. This concept of multiple different genes involved in the etiology of orofacial clefts is increasingly borne out by the results of genomic studies, as summarized below.

## Mapping genes for orofacial clefts

Different types of genetic studies create the opportunity to map genes in the absence of a well-defined model of inheritance. There are two general types of approaches: “linkage analysis” and “association analysis”. Linkage analysis always requires multiplex families and tests for co-segregation of observed genetic markers and a hypothetical gene controlling the affected vs. unaffected phenotype within a family, while association analysis simply tests for differences in frequencies of markers in samples of affected and unaffected individuals from a population, which could reflect linkage at a population level (termed “linkage disequilibrium”). Linkage analysis is a powerful approach for mapping individual genes for traits following clear Mendelian patterns within multiplex families, but it is less effective in mapping genes for complex traits (such as orofacial clefts).

Both linkage and association analysis can be used on one genetic marker or on many scattered over the entire genome. Importantly, both approaches can be adapted to accumulate evidence when multiple causal variants (in different genomic locations) exist in the data being analyzed (multiplex families or groups of cases and controls), and thus both can be used effectively to study orofacial clefts at the genome-wide level.

Numerous groups used linkage studies to map genes in multiplex cleft families ^23^, but limits on the number and size of these families and the underlying high level of “linkage heterogeneity” (where different families yielded evidence of co-segregation to different genes) made it extremely difficult to obtain consistent results. A consortium of multiple research groups pooling genome-wide markers (~400 microsatellite markers) identified linkage to six different chromosomal regions (chromosomes 1q32, 2p, 3q27–28, 9q21, 14q21–24, and 16q24) ^24, 25^. Further fine-mapping studies of these regions showed consistent evidence of linkage to chromosome 1q32 near the *IRF6* gene. Interestingly, mutations in *IRF6* account for most of the known cases of Van der Woude syndrome, a Mendelian autosomal dominant syndrome which includes orofacial clefts as a key phenotype ^23^. In addition, markers in and near this gene have also consistently shown evidence of association in population-based studies. Another gene, *FOXE1* on chromosome 9q21, yielded consistent evidence of linkage in multiplex families ^25, 26^ and recent association studies ^27, 28^. Thus, linkage studies based on multiplex families successfully identified some causal genes ^29^, but clearly multiple genes control risk to orofacial clefts and these vary from family to family.

Association studies ask a much more general question than linkage analysis, and typically this involves rather simple statistical comparisons of marker allele or genotype frequencies between groups of unrelated people. While interpreting a positive result from linkage analysis represents more compelling evidence that a putative gene controls risk, these multiplex families are rare (i.e. most cleft cases do not have any affected close relative) and individual families are not representative of the full population. Interpreting statistically significant results from an association study is more ambiguous and less definitive than results from linkage analysis, and unrecognized bias in the study design can produce spurious results (especially if the cases and controls are not drawn from the same population). As with linkage studies, one, several, or millions of markers can be compared between case and control groups, but of course as the number of tests increases some accommodation must be made for false positive results obtained by chance alone (i.e. where in truth there is no real difference between groups). Conversely, false negative results can become important too, i.e. where a key risk gene is missed because the markers used for tests of association are not informative and do a poor job of covering that gene in one population or another.

### Association studies of orofacial clefts

Association studies using case–control designs have been used to examine numerous selected “candidate” genes for orofacial clefts (CL/P and less commonly CP) for over 25 years ^30^, but the results were not always consistent across studies. This inconsistency could merely be because of limited sample sizes of individual studies, subtle biases in study design, or could reflect genetic differences across populations. Interestingly, sequencing the candidate genes themselves (and the regions immediately surrounding the gene) has identified some rare mutations that would impair the function of the corresponding gene product and could be directly causal ^31, 32^. While such rare variants are potentially biologically relevant and certainly justify further study of that candidate gene, their very low frequency in the population makes them difficult to incorporate either into standard statistical tests or for use in predicting risk to individuals (which is notoriously difficult for heterogeneous disorders).

Beginning early in the 21 ^st^ century and building upon the Human Genome Project, genome-wide association studies (GWAS) became feasible as genotyping technology improved to allow hundreds of thousands (or millions) of single nucleotide polymorphic (SNP) markers to be typed efficiently on large samples. The first GWAS of orofacial clefts used a classic case-control design to compare markers between CL/P cases from Germany to “universal” or unphenotyped German controls ^33^. The use of “universal” controls (typically adult controls from an independent study) is reasonable because the birth prevalence of orofacial clefts is relatively low. However, when conducting association studies, even subtle differences in the genetic background between the case and control groups can be a potential source of bias and must be considered. The key advantage of GWAS is its ability to identify simultaneously one or several regions of the genome likely to contain genes controlling risk to orofacial clefts. In the presence of multiple causal genes, there should be multiple genomic regions giving statistically significant results.

An alternative to the traditional case–control design is the family-based case–parent trio or “triad” design, which has the advantage of being robust to the confounding caused by “population stratification” or differences in marker allele frequencies between groups of cases and controls. Now the contrast is between the marker (allele, genotype, or haplotype) seen in the case and the set(s) that are possible given the parental mating type, yielding a comparison between the observed case and a matched set of “pseudo-controls” drawn from the parental mating type. This study design actually tests a composite null hypothesis of strict Mendelian transmission of marker alleles (or their genotypes), and rejecting this composite null represents evidence for both linkage and association ^34, 35^. This design was used by Beaty *et al*. ^36^ in an international collaboration supported by the Genes and Environment Association (GENEVA) consortium ^37^ where distinct racial groups (European and Asian) were well represented.

To date, there have been seven independent GWAS for CL/P and two for CP. They have identified at least two dozen different genes/regions achieving genome-wide significance for CL/P, but only one for CP. Leslie and Marazita ^38^ listed a dozen different genes/regions attaining genome-wide significance either in one GWAS or from meta-analysis across multiple GWAS, and Table 1 shows an updated list. Some of these genes have accumulated support from older candidate gene studies, and a few have been identified as causal genes for Mendelian malformation syndromes including orofacial clefts as a key phenotype. Of these genome-wide significant regions, four ( *IRF6* on 1q32–41, the gene desert region on 8q24, 17q22, and 10q25.3) appear to account for 20–25% of the estimated genetic variation in risk or “heritability” to CL/P, a much larger proportion of the estimated heritability attributable to markers identified by GWAS than seen for many other complex disorders ^17^.

However, the level of statistical support is frequently limited to one or another racial/ethnic group (e.g. the support for the gene desert region in 8q24 is consistently strong in European ancestry groups but much less so among Asian ancestry groups). This may reflect limitations in “tagging” by widely used SNP marker panels ^39^ or true differences in the frequency of causal mutations across populations. Because this strong statistical evidence comes from SNPs in a “gene desert” with no recognized coding regions nearby, it is particularly intriguing that mouse models have suggested this region may contain a regulatory region critical for normal craniofacial development ^40^.

## The future challenges: expanding into genomic sequencing

In many ways, the progression in genetic studies of orofacial clefts has repeatedly applied standard tools of genetics to a disorder that is unexpectedly complex and heterogeneous. Clearly there is a genetic basis to the etiology of orofacial clefts; however, it must involve multiple genes (maybe dozens) and several environmental risk factors (which may interact with different genes). Our analytical tools, both statistical and technological (for genotyping and sequencing), have greatly expanded in recent years but always seem to fall short of the mark in dealing with the high level of etiologic heterogeneity for this relatively rare disorder. Technological advances now make it feasible to sequence the entire exome (i.e. all gene regions known to code for proteins) and even the entire human genome. However, we still must carefully consider the most appropriate study design and how to analyze sequence data most effectively.

Family study designs can be very effective in identifying causal genes ^41^, but if many rare mutations can lead to the same phenotype, it is entirely possible that each family could reflect effects of a unique gene or unique combination of genes. Bureau *et al*. ^42, 43^ recently showed how whole exome sequencing (WES) data in distant affected relatives (second-degree and more distant relatives) drawn from multiplex families originally recruited for linkage studies could be used to identify rare variants that modify risk to orofacial clefts. From a long list of candidate genes, this approach identified one novel variant in *CHD1* shared by three affected second cousins in a single multiplex family. *CHD1* is a strong candidate gene because mutations in this gene can cause both gastric cancer and orofacial clefts, and several studies have shown evidence of association or excess of rare variants in this gene ^44– 47^. A comprehensive whole exome analysis revealed a separate novel variant in *ADAMTS9* achieving statistical significance over the entire exome in a small number of families ^42^. While this gene had been identified as potentially causal in mouse models, this is the first evidence from human studies, and the risk allele is extremely rare. This same method of testing for rare shared variants can be applied to structural variants, such as deletions detected from WES data ^48^.

Leslie *et al*. ^49^ demonstrated the potential of sequencing studies in a large study of case–parent trios (where 13 regions around candidate genes were sequenced). This case–parent trio design allows the detection of *de novo* mutations, where a new mutation (not present in the parents) occurs in the child. This type of mutation simply cannot be identified in case–control studies, and such mutations are by their nature extremely rare. Also, if a *de novo* mutation lies outside any known coding or regulatory region, it is difficult to judge its true functional status. This group concluded *de novo* variants probably don’t represent a major etiologic component for orofacial clefts, although they may be relevant for other complex diseases.

This case–parent trio study of sequencing data yielded evidence consistent with previous GWAS studies, where multiple genomic regions showed strong statistical evidence of linkage and association for common polymorphic markers, but it also was able to analyze rare variants that could themselves be directly causal for orofacial clefts. Careful analysis of low-frequency and rare variants using available methods for collapsing or combining multiple variants within a gene to assess the overall impact of that gene on risk yielded mixed results: two of 13 candidate regions ( *NOG* and *NTN1*) showed some statistical evidence for distinct rare variants within the gene being important in controlling risk, while four of the 13 genes yielded no such evidence ( *BMP4*, *FGFR2, MSX1*, and *PTCH1*). Because obtaining statistical evidence for rare variants is always difficult, even in large studies, building a biological case for a putative causal mutation requires functional studies for confirmation ^49^.

Large-scale whole genome sequencing (WGS) studies are now underway and are being applied to a number of complex and heterogeneous diseases (including orofacial clefts). Practical challenges in using WGS data include cleaning and processing the huge volume of data. The amount of data scales up dramatically from GWAS to WES to WGS: GWAS data represent hundreds of thousands or millions of typed markers (and potentially millions more imputed markers); WES represents 1–2% of the human genome sequenced 30–50 times and aligned to yield a consensus sequence for ~50 Mb of the genome; WGS represents similarly aligned read data on all 30 billion base pairs of the genome (although even with WGS there are gaps and some regions are not well covered). The primary advantage of WGS data is that it can be used to analyze any type of sequence variant from the reference genome (single nucleotide variants or SNVs, including the common SNP markers), small deletions (termed indels), or larger structural changes (e.g. deletions and duplications, which are collectively called copy number variants or CNVs, plus more complex structural variants such as inversions and translocations where the order of genes may be changed through chromosomal breakage and exchange). The challenge comes in defining the particular assessment/comparison being made with variants identified by WGS data (which is always a reflection of the study design and hypothesis being considered), and then interpreting any “significant” differences from expected. The study design always determines the hypothesis to be tested (strict Mendelian transmission, co-segregation, differences in frequencies, etc.), but the huge number of tests must be considered, along with the potential biases inherent in the study design. Dealing with WGS data will involve tens of millions of markers or SNVs on individuals, and most of these will be quite rare. Most observed sequence variants will occur outside known coding regions, and our ability to recognize important regulatory regions remains limited.

The more important challenge facing genetic studies of orofacial clefts is to develop strategies for dealing with multiple causal genes within any data set and applying these strategies effectively to orofacial clefts as a complex and heterogeneous birth defect. The full range of study designs (selected relatives from large multiplex families, nuclear families, case–parent trios, and groups of unrelated individuals) is available, but interpreting the biological function of a novel variant detected by sequencing will not be simple. Furthermore, because any given patient could represent the effects of one or more variants in several different genes, translating new genetic findings into meaningful medical or public health applications remains a daunting task. In reality, for orofacial clefts (or any other birth defect), because the first occurrence occurs well before birth, it is difficult to define opportunities to intervene. Nonetheless, the tools to detect genes controlling risk to orofacial clefts have expanded greatly in this first part of the 21 ^st^ century, and it is our challenge to use them as effectively and efficiently as possible. It is likely that there will be classes of “causal genes” and “genetic risk factors”: a few genes with “major effects” may simultaneously control truly Mendelian malformation syndromes and show association with apparent non-syndromic forms of orofacial clefts, while possibly many “genetic risk factors” have the typical modest estimated effect sizes identified through GWAS but nonetheless achieve genome-wide significance as studies are combined via meta-analysis.

## Abbreviations

CI, confidence interval; CL, cleft lip; CL/P, cleft lip with or without cleft palate; CLP, cleft lip and palate; CP, cleft palate; GWAS, genome-wide association study; GxE, gene–environment; SNP, single nucleotide polymorphism; SNV, single nucleotide variant; WES, whole exome sequencing; WGS, whole genome sequencing.
